# Informing future policy for trauma prevention: The effect of the COVID-19 ‘National state of disaster lockdown’ on the trauma burden of a tertiary trauma centre in the Western Cape of South Africa

**DOI:** 10.1016/j.afjem.2021.06.002

**Published:** 2021-07-07

**Authors:** Scott H. Mahoney, Elmien Steyn, Hendrik Lategan

**Affiliations:** Tygerberg Hospital, Division of Surgery, Faculty of Medicine and Health Sciences, Stellenbosch University, Cape Town, South Africa

**Keywords:** Trauma, Prevention, COVID-19, Lockdown, Injury

## Abstract

**Introduction:**

Strategies to reduce the burden of trauma are not only a global priority, but also a South African public health priority due to a disproportionately large trauma burden. Identification of the contributors to preventable injuries would assist in guiding policy and prevention strategies at a local and international level. In response to SARS-nCOV-2 (COVID19), a national restrictive lockdown was implemented in South Africa with, amongst other restrictions, a complete ban on non-essential travel and alcohol sales. With the most intensive restrictions implemented between March to May 2020, this period offers an unprecedented opportunity for the assessment of social restrictions on possible effects of trauma burdens.

**Methods:**

A retrospective chart review was conducted between March to May 2019 and compared to data from the same period in 2020. Descriptive analysis was undertaken to understand the influence of lockdown on demographics and injury causation in trauma presentations.

**Results:**

The results showed a 51.42% decline in trauma during the early lockdown period. Sub-analyses however, revealed little change in the mechanism of injury ratios and the demographics of presenting patients.

**Conclusion:**

This study shows that although all cause presentation of trauma cases was reduced following the implementation of lockdown procedures in 2020, the injury patterns and ratios of intentional to accidental harm remained largely unchanged. This prompts the need for further research and root cause analysis into how trauma prevention strategies can be improved. This will assist with the improved efficacy of trauma prevention policies in a country with a well-documented trauma burden and thus a pressing need for an implementable and nationwide harm reduction policy.

## African relevance

• Africa has a high burden of disease and reduction of preventable injury can assist with financial and social implications that injuries have on development.
• Primary prevention through policy frameworks can be cost effective in reducing trauma burdens in disadvantaged and low/middle income settings.
• This research can be extrapolated to many African countries with similar socio-economic circumstances and thus contributes to the body of science in trauma prevention research.
• Reduction of trauma burdens assists with capacity and resource allocation to treat COVID cases in a resource limited setting.

## Introduction

South Africa is a diverse and developing upper-middle-income country [1] with a well-documented trauma burden [2]. Trauma is one of the leading causes of mortality and morbidity in young adult South Africans and results in a significant strain on the public health system as well as the socio-economic well-being of patients and their families. The Western Cape province of South Africa is known for its high prevalence of interpersonal violence as well as an expansive socio-economic divide [3].

Due to a growing population, particularly in the Western Cape, pressure on the health care system has increased. All levels of healthcare services in the Western Cape are affected by high volumes of injuries, ranging from interpersonal violence to transport-related incidents. The Western Cape Burden of Disease report indicated a near 23% increase in the population of the Western Cape from approximately 5,5 million to nearly 7 million people between 2009 and 2019 [4]. The subgroup with the largest population growth is the 20–35 years of age group. Notably, the most common age group presenting with trauma secondary to interpersonal violence is the age group 18–40 years [2].

A significant increase in gun-related injuries amongst men was documented, with a doubling of gun-related homicides evident between 2010 and 2016. Blood alcohol levels were notably increased in these victims, with 45% of victims having levels more than double the legal driving limit [4].

Tygerberg Hospital (TBH) is the main academic facility for Stellenbosch University and serves as the referral centre for a large rural, semi-urban and urban population. TBH's Trauma Centre functions as a Level 1 (tertiary referral) centre with 12,000–15,000 seriously injured persons per year.

In response to the first local cases of SARS-nCOV-2 (COVID19), the South African government declared a State of Disaster and instituted a national restrictive lockdown with a night-time curfew and a complete ban on non-essential travel and alcohol transport and sales, enforced by members of the military and police services [5]. Citizens were required to stay home unless they were deemed to be a part of an essential service or requiring access to food or medical care. The purpose was to “flatten the curve” and enable the optimisation of the public health system in preparation for the pandemic.

During this lockdown period, restrictions on civilian movement substantially reduced both road traffic activity and pedestrian activity on or near roads. Access to substances such as alcohol was also eliminated, with all liquor vendors forced to close during the initial lockdown period. The nationwide enforced restrictions commenced on the 27th March 2020 and brought about a significant reduction in the incidence of trauma cases presenting to emergency centres [6]. The ban on alcohol sales and some travel restrictions was lifted on 31 May 2020.

These national interventions provided an opportunity to investigate the effect of stringent alcohol prohibition, reduced interpersonal encounters, and travel restrictions on trauma patterns at TBH. The aim of this study was to assess the effect of the COVID19 lockdown on the nature and presentation of trauma cases presenting to TBH by comparing patient volumes and injury patterns over two time periods with and without lockdown measures in place. Further objectives included gathering information that will contribute to the discussion on how measures deployed during this lockdown period may be utilised in future as strategies to reduce the burden of trauma in South Africa.

## Methods

A retrospective review of the registry of patients presenting to Tygerberg Hospital's trauma unit was conducted over two time periods, 1st March to 31st May 2019 and 1st March to 31st May 2020. This allowed for data from two comparable periods to be collected: the time period without lockdown in 2019 and the period during lockdown of 2020. Sub-analyses of variables including demographics, mechanism of injury and causation of injury were done from 1st of April to 31st May for the two different years.

All patients self-presenting or referred to the trauma unit within the study periods were included in the study. No patients were excluded from the study. Data were collected from the trauma registry as well as the clinical notes and anonymised according to the requirements of the study protocol. The patient registry is the most consistent and reliable data source as patients cannot be seen in the unit without being captured in the register. The register records the patient demographics, as well as time and date of presentation. It also captures the mechanism of injury and the location of the injury on the patient's body. These variables were capture for analysis on a pre-designed data matrix using Microsoft® Excel® Version 16. Data were subjected to a descriptive analysis using SPSS Statistics for Windows®, version 26.0 (IBM Corp.®, USA) with multiple sub-analyses using either Fischer exact test or Pearson Chi-Square test depending on the number of data points to be analysed. Statistical significance was set at *p* < 0.05 for sub-analysis. The Mann-Whitney *U* test was used for non-parametric data analysis with a *p*-value set at *p* < 0.05.

Ethics approval was obtained from the Stellenbosch University's Health Research Ethics Committee (SunHREC) N20/07/037-COVID-19. The study was also registered with the National Health Research Database (NHRD) WC_202007_036.

## Results

**Fig. 1:**
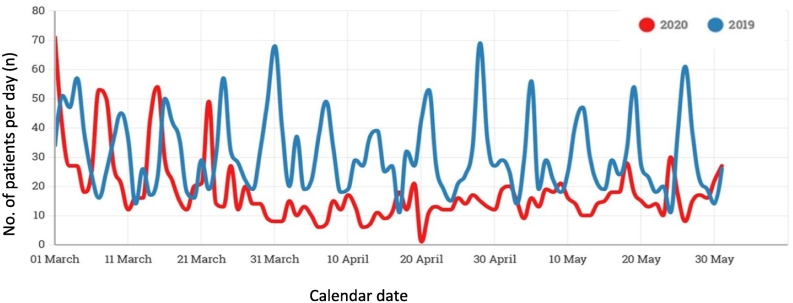
Daily case volume at TBH Trauma Centre for the period March to May 2019 (blue) and 2020 (red). (For interpretation of the references to colour in this figure legend, the reader is referred to the web version of this article.)

A total of 4505 patient cases were captured during the study periods, comprised of 2834 patients over the 2019 period and 1671 over the 2020 period. It is important to note that total patient numbers presenting during the full calendar months of the March to May periods were compared to identify the possible drop off in case load after lockdown. However, as lockdown commenced on 27 March 2020, comparing March 2019 to March 2020 showed little difference in trends (Fig. 1). To enable a more accurate descriptive analysis, the comparison between 2019 and 2020 was thus limited to the April to May periods.

In a sub-analysis of the two study periods, April to May 2019 (*n* = 1797) and April to May 2020 (*n* = 872 patients), a 51.42% decrease in total case load was noted in the 2020 period. A clear and sudden decline was noted in daily case presentations correlating with the commencement of the lockdown period on the 27th of March 2020, however persistent temporal peaks over weekends remained common in both study periods (Fig. 1).

Age categorisation was done according the United Nation's Global population age structures [7] comparing the 2019 to 2020 periods and showed no significant difference in the age demographic (Table 1). Age was non-normally distributed with a median age of 29 years and an interquartile range of 18 and 19 for 2019 and 2020 respectfully. No significance difference between the 2019 and 2020 groups was noted when analysed with the Mann-Whitney *U* test (*p*-value = 0.671).

**Table 1:** Age distribution of patients treated in TBH trauma centre between April–May 2019 and 2020.

Age category		Year grouping		Combined Total
		2019	2020	
0–14 (children)	Count	253	132	385
	% within Year grouping	14,1%	15,1%	14,4%
15–24 (youth)	Count	375	179	554
	% within Year grouping	20,9%	20,5%	20,8%
25–64 (working age)	Count	1082	525	1607
	% within Year grouping	60,2%	60,2%	60,2%
65+ (older persons)	Count	87	36	123
	% within Year grouping	4,8%	4,1%	4,6%
Total	Count	1797	872	2669
	% within Year grouping	100,0%	100,0%	100,0%

Gender comparison between the 2019 (Male – 76.2%; Female – 23.8%) and 2020 groups (Male – 77.2%; Female – 22.8%) revealed no significance shifts in the two study periods (*p*-value = 0.585).

Sub-analysis of mechanism of injury patterns between the two data sets showed little significant changes between the 2019 and 2020 groupings. Although there was a definitive decrease in case numbers of most mechanisms of injury between 2019 and 2020, burns and accidental penetrating trauma saw an absolute rise in patient numbers during the two periods assessed.

When assessing the proportion of each mechanism, an increase was noted in the ratio of patients with gunshot wounds (11.5% to 15.3%) as well as assault with blunt trauma (13.1% to 20.9%). Expectedly, the proportion of motor-vehicle collision (MVC) cases nearly halved from 15. 0% to 8.4%, however pedestrian vehicle collision (PVC) remained similar from 4.4% to 4.8%. No cases of train incidents were received in the unit over the 2020 period (Table 2).

**Table 2:** Sub-analyses of mechanism of injury for April–May 2019 and 2020.

		2019	2020
Assault - penetrating knife	Count	416	102
	% within Year grouping	23.1%	11.7%
Assault - penetrating GSW	Count	206	133
	% within Year grouping	11.5%	15.3%
Assault - blunt trauma	Count	236	182
	% within Year grouping	13.1%	20.9%
MVC	Count	270	73
	% within Year grouping	15.0%	8.4%
PVC	Count	79	42
	% within Year grouping	4.4%	4.8%
Accidental-fall	Count	323	199
	% within Year grouping	18.0%	22.8%
Accidental - penetrating trauma	Count	18	24
	% within Year grouping	1.0%	2.8%
Accidental - Blunt trauma	Count	99	52
	% within Year grouping	5.5%	6.0%
Burns	Count	13	16
	% within Year grouping	0.7%	1.8%
Dog Bite/envenomation	Count	12	10
	% within Year grouping	0.7%	1.1%
Unknown Mechanism of Injury	Count	109	32
	% within Year grouping	6.1%	3.7%
Self-harm	Count	9	7
	% within Year grouping	0.5%	0.8%
Train incident	Count	7	0
	% within Year grouping	0.4%	0.0%

Mechanisms of injury were then categorised for each patient into causation of injury, chiefly, whether intentional harm or accidental harm (Table 3). Again, no significant differences were noted between 2019 and 2020 (*p*-value = 0.574).

**Table 3:** Sub-analyses of causation of injury for 2019 and 2020.

Causation of Injury	Year grouping			Total
	2019	2020		
Intentional	Count	858	417	1275
	% within Year grouping	47.7%	47.8%	47.8%
Accidental	Count	830	423	1253
	% within Year grouping	46.2%	48.5%	46.9%

With the knowledge that there is no significant difference in injury mechanisms between study periods, data was combined to show the trends in the injury patterns and demographics of the trauma units patient load effectively over a 6-month period.

Working age patients (25–64 years) represented 60.2% of all patients and more than three quarters of patients were male. A 2019 and 2020 grouped sub-analysis of categorised day of the week in relation to causation of harm showed increased intentional harm (46.2% to 57.4%) from weekday (Monday to Friday) to weekend periods (Saturday to Sunday) (Table 4). A predominance of accidental harm was shown over weekdays (53.8%) when compared to weekends (42.6%).

**Table 4:** Cross-tabulation of categorised day of week to causation of harm.

Categorised days of the week		Intentional	Accidental
Monday to Friday	Count	723	843
	% within Days of week Mon-Fri & Sat-Sun	46.2%	53.8%
Saturday to Sunday	Count	552	410
	% within Days of week Mon-Fri & Sat-Sun	57.4%	42.6%

## Discussion

There is a substantially high overall injury death rate in South Africa of 157.8 per 100,000 population [8], which is close to twice the global average. Trauma is therefore a leading cause of mortality and lost disability-adjusted life years [8] and prevention of injuries should be prioritised to reduce the impact of trauma on our healthcare system and affected population groups. Multiple programs have already been rolled out to target high risk groups and reduce interpersonal violence including the Western Cape Alcohol-Related Harms Reduction Policy [9].

During the restrictive period of the national lock-down in South Africa, the trauma burden at a tertiary trauma unit in the Western Cape was reduced by over 50%. However, the lack of significant change in proportions of mechanisms of injury was surprising. Furthermore, the ratio of intentional to accidental harm remained consistent throughout both periods. Further investigation into the factors that drive this would be most beneficial to assisting in building trauma prevention strategies.

South Africa's strictest period of lockdown was implemented on the 27th of March 2020 and provided an opportunity to investigate the effect of multiple social interventions on trauma burdens across the country's health services. Many facilities throughout South Africa reported drastic reductions in emergency centre patient loads, mostly reported from district and regional centres. Morris et al. reported a 47% reduction of trauma burden at a regional hospital in Kwa-Zulu Natal with no significant change in the varying severity of patient injuries following the commencement of lockdown [6]. By comparison, a district hospital within the Cape Town Metropolitan area only reported a 20% reduction in trauma related cases [10]. Another tertiary and more comparable trauma unit in the Western Cape metropole recorded a 53% reduction in patients seen during the initial lockdown period [11]. *Navsaria et al* also reported a reduction of road traffic injuries in keeping with the trends noted at TBH [11].

Previous episodes of respiratory virus outbreaks such as the Severe Acute Respiratory Syndrome (SARS) outbreak in 2003, were clearly associated with decreased trauma presentations, as reported by Huang et al. who noted a 57.6% decline in trauma presentations to their Taiwanese emergency centre in 2003 [12]. However, the authors noted that this was largely associated with public fear in attending medical facilities rather than an enforced lockdown period.

Although in most sub-analyses there was little significant difference between the 2019 and 2020 groups, noteworthy trends in trauma injury patterns within our setting can be drawn to inform preventative action policies. Injury prevention strategies with proven efficacy include visible policing in high-risk areas and reduced access to alcohol. Weekend clustering of injuries is certainly an area of focus which could be directed to law enforcement agencies by engaging them and the communities within which they operate. The accumulated evidence should be utilised to increase visible policing in specifically high risk areas to primarily reduce interpersonal violence and lawlessness [13,14].

Combined with the knowledge that working-class men predominate when it comes to trauma presentations, more focus should be placed on this demographic through social and wellness interventions. Although the available data provided no proven prevalence of alcohol or illicit substance use in the patients encountered, it is well documented that binge drinking is common amongst males over the age of 15 [15]. Furthermore, Forensic Pathology Services report that 50% of homicide victims had detectable alcohol levels post-mortem and 45% of those were over the South African legal driving limit [16]. Thus, implementation of the alcohol harm reduction policy and integrated violence prevention policy framework is imperative to reduce the impact that disinhibition by alcohol has on the incidence of trauma (remembering that both intentional and non-intentional trauma are affected by alcohol) [17,18]. These policies aim to decrease the ease of access to alcohol. They also aim to boost alcohol related education as part of their strategy, specifically over higher consumption periods such as weekends.

Although extensive data were collected, there are limitations in the interpretation of results. The study was conducted in a single tertiary level facility in the Western Cape; therefore, the findings may not be relevant in different settings. Additional data from multiple sites and varying levels of care may increase the relevance of the study. It was not possible for study purposes to retrieve all patient files retrospectively due to the ongoing occupational health and safety COVID restrictions, accounting for an inflated number of “unknown” data points and limiting available data variables.

Studies such as these are valuable for advocacy, showing the need for specific interventions and general socio-economic upliftment. Trauma prevention strategies should be developed in collaboration with affected communities, local clinicians, researchers and political role players for the benefit of the general population.

COVID-19 has had a substantial global effect on the public healthcare systems. The nationwide lockdown implemented to preserve capacity in the South African healthcare system resulted in a dramatic 51.42% reduction in injury presentations at TBH, indicating a fortuitous window of opportunity to create and implement more effective trauma prevention strategies.

This study revealed relative consistency in injury patterns and patient demographics despite a drop in overall numbers. The highest risk group for traumatic injuries is working class men, who, when injured or killed, burden not only the health care system, but also cause a socio-economic ripple effect in communities and the workplace. The complex epidemiology of traumatic injuries in the Western Cape requires more emphasis on innovative preventative strategies to reduce the trauma burden.

The evolving lockdown levels utilised by government may provide an opportunity for further investigation into the effect of specific social interventions on trauma burdens at local facilities. Further research and reporting of trauma injury patterns and epidemiology should be encouraged at healthcare facilities to aid in future development of trauma prevention strategies.

## Dissemination of results

Results have been distributed by the authors to the institution wherein the study took place. The results have been utilised at various managerial levels to assist with planning and provision of healthcare services.

## CRediT authorship contribution statement

Authors provided various contributions to this research. Conceptualisation and development of the protocol; SHM 60%, ES 20%, HL 20%. The acquisition and analysis of data; SHM 80%, ES 10%, HL 10%. Revision and compilation of the manuscript; SHM 70%, ES 15%, HL 15%. Approval for the publication of the final manuscript was received form all authors.

## Declaration of competing interest

The authors declare no conflicts of interests.
